# QM quality atomic charges for proteins

**DOI:** 10.1186/1758-2946-6-S1-P61

**Published:** 2014-03-11

**Authors:** Stanislav Geidl, Crina-Maria Ionescu, Radka Svobodová Vařeková, Jaroslav Koča

**Affiliations:** 1 National Centre for Biomolecular Research, Faculty of Science and CEITEC - Central European Institute of Technology, Masaryk University, Brno, 625 00, CZ, Czech Republic

## 

The concept of atomic point charges is well established in theoretical chemistry. Atomic point charges have played an important role in understanding and modeling chemical behavior by allowing to extract and quantify information stored in the molecular electron distribution of chemical compounds. Thus, atomic point charges have been used to estimate reactivity indices, dissociation constants, partition coefficients, the electrostatic contribution in molecular dynamics or docking studies. It is therefore desirable to have knowledge of the values of atomic charges in proteins (see, e.g., [1]). Unfortunately, accurate and universally applicable approaches for atomic charge calculation based on quantum mechanics (QM) are very time consuming and thus cannot be employed for large biomolecules like proteins. An alternative is to use empirical charge calculation methods, such as the electronegativity equalization method (EEM) [2], which is very fast and has accuracy comparable to QM. The challenge is to calibrate (i.e., parametrize) this method for proteins. This parameterization can be done using atomic charges calculated by different types of QM approaches. EEM can be as accurate as the QM approach for which EEM was calibrated.

In our work, we present the workflow of the EEM calibration process. Afterwards, we calibrate and validate EEM models for 12 types of QM charges, including the newest approaches like iterative Hirshfeld [3]. The accuracy of the obtained EEM models is evaluated on insulin and ubiquitin. We also show two case studies demonstrating the applicability of atomic charges computed via EEM: a small docking study, and the calculation of electrostatic potential based on the EEM charges [4].
